# Reliability and validity of a brief sleep questionnaire for children in Japan

**DOI:** 10.1186/s40101-017-0151-9

**Published:** 2017-09-15

**Authors:** Masakazu Okada, Shingo Kitamura, Yoshitaka Iwadare, Hisateru Tachimori, Yuichi Kamei, Shigekazu Higuchi, Kazuo Mishima

**Affiliations:** 1 Department of Kansei Science, Graduate School of Integrated Frontier Science, Kyushu University, 4-9-1 Shiobaru, Minami-ku, Fukuoka, 815-8540 Japan; 2 Department of Psychophysiology, National Institute of Mental Health, National Center of Neurology and Psychiatry, 4-1-1 Ogawa-Higashi, Kodaira, Tokyo, 187-8553 Japan; 3 Department of Child and Adolescent Psychiatry, National Center of Global Health and Medicine, Kohnodai Hospital, 1-7-1 Kohnodai, Ichikawa, Chiba 272-8516 Japan; 4 Department of Mental Health Policy and Evaluation, National Institute of Mental Health, National Center of Neurology and Psychiatry, Kodaira, Tokyo, 187-8553 Japan; 5 Department of Human Science, Faculty of Design, Kyushu University, 4-9-1 Shiobaru, Minami-ku, Fukuoka, 815-8540 Japan

**Keywords:** Sleep questionnaire, Reliability, Validity, Children, Sleep problems

## Abstract

**Background:**

There is a dearth of sleep questionnaires with few items and confirmed reliability and validity that can be used for the early detection of sleep problems in children. The aim of this study was to develop a questionnaire with few items and assess its reliability and validity in both children at high risk of sleep disorders and a community population.

**Methods:**

Data for analysis were derived from two populations targeted by the Children’s Sleep Habits Questionnaire (CSHQ): 178 children attending elementary school and 432 children who visited a pediatric psychiatric hospital (aged 6–12 years). The new questionnaire was constructed as a subset of the CSHQ.

**Results:**

The newly developed short version of the sleep questionnaire for children (19 items) had an acceptable internal consistency (0.65). Using the cutoff value of the CSHQ, the total score of the new questionnaire was confirmed to have discriminant validity (27.2 ± 3.9 vs. 22.0 ± 2.1, *p* < 0.001) and yielded a sensitivity of 0.83 and specificity of 0.78 by receiver operator characteristic curve analysis. Total score of the new questionnaire was significantly correlated with total score (*r* = 0.81, *p* < 0.001) and each subscale score (*r* = 0.29–0.65, *p* < 0.001) of the CSHQ.

**Conclusions:**

The new questionnaire demonstrated an adequate reliability and validity in both high-risk children and a community population, as well as similar screening ability to the CSHQ. It could thus be a convenient instrument to detect sleep problems in children.

## Background

Recent evidence suggests that sleep disorders and sleep behavior problems (i.e., evening-type lifestyle and sleep deprivation) are common in children [1–4]. Such conditions affect between 25 and 45% of preschool and school-aged children and adolescents and are associated with behavioral deficits and impaired mental functions at home and/or at educational institutions [5–7]. Because there are a small number of sleep laboratories and pediatric psychiatric hospitals, it has become important to develop simple instruments that can be used by primary care physicians, public health nurses, teachers, and parents/guardians for early detection of sleep problems in children, particularly in those at high risk of sleep disorders in a community setting.

A questionnaire is a useful tool to screen for sleep problems in children, and a number of pediatric sleep questionnaires have been developed [8]. The questionnaire most frequently used for children is the Children’s Sleep Habits Questionnaire (CSHQ) [9], which is widely used for both clinical and research purposes.

The CSHQ has been used for children in various settings and with a wide range of ages. It has been used in a number of clinical and epidemiologic studies to examine sleep behaviors and sleep problems in children with sleep disorders [10], developmental disorders [11], and anxiety disorders [12] and in the general pediatric population [13, 14]. Because the CSHQ is used for both school-aged and preschool children [15], it would be valuable to reduce the number of items in the CSHQ and develop a simpler questionnaire with similar screening ability. Accordingly, this study aimed to develop a simplified sleep questionnaire based on the CSHQ but with fewer items and to assess its reliability and validity in children at high risk of sleep disorders and in a community population.

## Methods

### Participants and settings

Participants comprised both the parents of 432 new outpatients aged 6–12 years recruited at the Department of Child and Adolescent Psychiatry, Kohnodai Hospital, National Center for Global Health and Medicine, and the parents of 178 students from a previous school-based community study [14]. The survey of the community population, conducted in November 2009, involved the parents of 330 students aged 6–12 years (first to sixth graders) enrolled in public elementary schools. The details of the survey have been published elsewhere [14]. In brief, after the parents gave informed consent, they were asked to answer the CSHQ. A total of 296 questionnaire sheets were returned after 1 week (response rate, 89.7%). In this study, we excluded 118 surveys—1 because it was missing age and sex information and 117 because they were missing at least one of the CSHQ items (meaning that the total score could not be calculated)—and used 178 surveys from the community sample with responses to all items of the CSHQ.

The data of the clinical population were collected between July 2008 and March 2015 from the parents of patients aged 1–15 years. In total, the CSHQ was administered to 1967 parents; in this data set, complete data were obtained for 432 children with no history of psychotropic drug administration.

This study was approved by the Ethics Committee of the National Center for Global Health and Medicine, Japan, and the Institutional Review Board of the National Center of Neurology and Psychiatry, Japan.

### Development of a brief sleep questionnaire for children from the CSHQ

The CSHQ, developed by Owens et al. [9], is a retrospective 52-item questionnaire for children. Parents or guardians are asked to respond to all items by recalling the sleep behavior of their children over a typical recent week. Items are rated on a 3-point scale; a higher score indicates more frequent occurrence of sleep problems. In the questionnaire, 33 items are used to calculate total score of the CSHQ and are grouped into eight subscales—Bedtime Resistance (6 items), Sleep Onset Delay (1 item), Sleep Duration (3 items), Sleep Anxiety (4 items), Night Wakings (3 items), Parasomnias (7 items), Sleep Disordered Breathing (3 items), and Daytime Sleepiness (8 items). The cutoff score with the best diagnostic confidence is reported as 41. The Japanese version of the CSHQ has already been developed [16]. One of the authors (K.M.) empirically extracted the 19 items of our new questionnaire from the 52 items of the Japanese version of the CSHQ by focusing on (1) sleep problems highly prevalent in children and (2) clinically important sleep problems in children, based on his clinical experience. Item selection was confirmed by a number of sleep medicine specialists and pediatric psychiatrists belonging to the National Center for Global Health and Medicine, Japan, or the National Center of Neurology and Psychiatry, Japan. The 19 finally selected items are shown in Table 1. Of these, 4 items were related to “Bedtime Behavior,” 9 items were related to “Behavior During Sleep,” 5 items were related to “Difficulty with Morning Waking,” and 1 item was related to “Hypersomniac Symptoms.” As in the CSHQ, the items were evaluated using a 3-point Likert scale (1 = *rarely* [never or once per week], 2 = *sometimes* [two to four times per week], and 3 = *usually* [five or more times per week]).

**Table 1:** Items of the short version of the sleep questionnaire for children

				I-T correlation	
		Mean	SD	*r*	*p*
Bedtime behavior					
1.	Child falls asleep with rocking or rhythmic movements	1.17	0.50	0.174	0.000
2.	Child needs special object to fall asleep (doll, special blanket, etc.)	1.40	0.77	0.216	0.000
3.	Child resists going to bed at bedtime	1.49	0.67	0.332	0.000
4.	Child is afraid of sleeping in the dark	1.55	0.82	0.234	0.000
Sleep behavior					
5.	Child talks during sleep	1.41	0.58	0.232	0.000
6.	Child is restless and moves a lot during sleep	1.68	0.77	0.345	0.000
7.	Child sleepwalks during the night	1.04	0.21	0.185	0.000
8.	Child grinds teeth during sleep (your dentist may have told you this)	1.29	0.55	0.247	0.000
9.	Child snores loudly	1.28	0.54	0.205	0.000
10.	Child seems to stop breathing during sleep	1.04	0.20	0.147	0.000
11.	Child snorts and/or gasps during sleep	1.03	0.20	0.187	0.000
12.	Child awakens during night screaming, sweating, and inconsolable	1.07	0.29	0.224	0.000
13.	Child awakens alarmed by a frightening dream	1.10	0.31	0.262	0.000
Difficulty with morning waking					
14.	Child wakes up in negative mood	1.66	0.77	0.404	0.000
15.	Child has difficulty getting out of bed in the morning	1.87	0.84	0.467	0.000
16.	Child takes a long time to become alert in the morning	1.76	0.82	0.492	0.000
17.	Child wakes up very early in the morning	1.32	0.59	− 0.142	0.000
18.	Child has a good appetite in the morning	1.79	0.78	0.189	0.000
Hypersomniac symptoms					
19.	Child suddenly falls asleep in the middle of active behavior	1.04	0.21	0.066	0.102

### Reliability

The reliability of the new questionnaire for the community, clinical, and combined samples was assessed for internal consistency using Cronbach’s alpha coefficients. We evaluated the internal consistency based on Cortina et al. [17].

### Validity

Discriminant validity was assessed by comparing the total score of the new questionnaire between the sample with a total CSHQ score of ≥ 41 and that with a total score < 41 and using the receiver operator characteristic (ROC) curve to test the cutoff value of the CSHQ. The area under the curve, sensitivity, and specificity of the new questionnaire were determined. Concurrent validity was investigated by Pearson’s correlation coefficients between total score of the new questionnaire and that of the CSHQ. We also assessed the correlation between the new questionnaire and the scores of each subscale of the CSHQ.

**Fig. 1:**
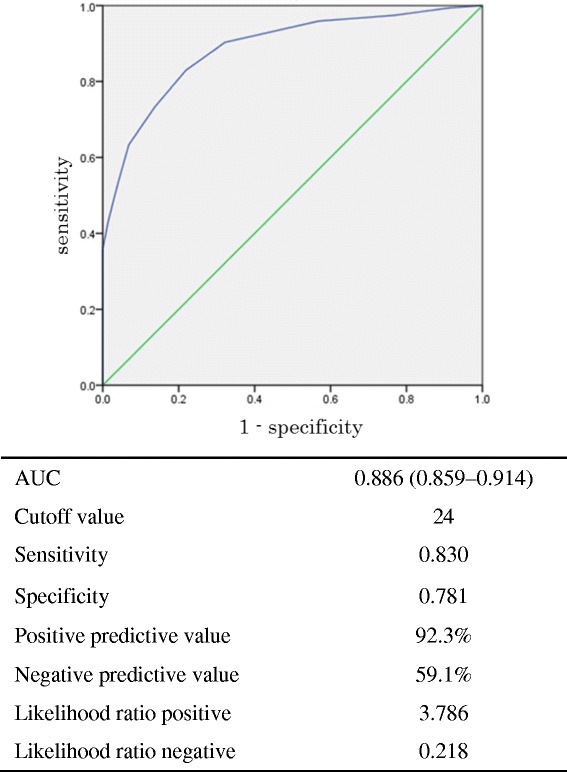
ROC curve for the total score of the new questionnaire using the CSHQ cutoff score (≥ 41) as a threshold

**Fig. 2:**
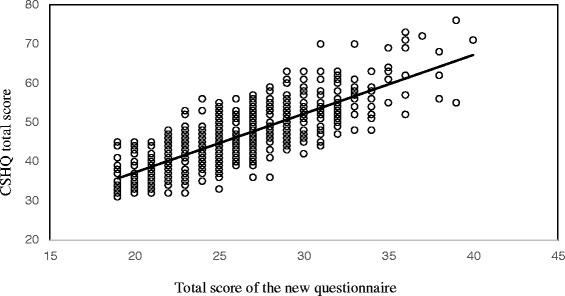
Correlation between the new questionnaire and the CSHQ for the total score

### Comparison with the model obtained by model selection

We compared the new questionnaire to a statistically reduced model. This model was mathematically selected by using Akaike’s information criterion (AIC), which is a model selection criterion based on goodness of prediction, to reduce the number of items from the CSHQ. After the logistic regression analysis of the 52 items of the CSHQ using a random selection of half of the combined samples, we adopted the backward/forward stepwise selection procedure to obtain the model that minimized the AIC value using the stepAIC function in the MASS package for R. We then confirmed the reliability and validity of the selected model by using the other half of the combined samples.

### Statistical analyses

Statistical analyses were performed using IBM SPSS Statistics 21.0 (IBM Corporation) and EZR (Saitama Medical Center, Jichi Medical University), which is a graphical user interface for R (The R Foundation for Statistical Computing, version 2.13.0) [18], based on a modified version of R commander (version 1.6-3).

## Results

### Participant characteristics

The combined sample provided data on 610 children—376 boys (61.6%) and 234 girls (38.4%)—with a mean age of 9.0 ± 1.8 years. The mean value of the CSHQ total score was 46.2 ± 7.8, with 464 children (76.1%) scoring above the CSHQ cutoff. The community and clinical samples differed according to age, sex, and CSHQ total score. The community sample was significantly older (9.3 ± 1.8 vs. 8.8 ± 1.8 years, *p* = 0.003) and had a higher proportion of girls (50.6 vs. 33.3%, *p* < 0.001). In addition, the clinical sample had a significantly higher CSHQ total score (47.5 ± 8.1 vs. 42.9 ± 6.1, *p* < 0.001) and a significantly higher proportion of children who scored above the CSHQ cutoff (81.7 vs. 62.4%, *p* < 0.001). The diagnoses based on the DSM-IV in the clinical sample were pervasive developmental disorders (*n* = 139, 32.2%), attention-deficit and disruptive behavior disorders (*n* = 114, 26.4%), anxiety disorders (*n* = 39, 9.0%), depressive disorders (*n* = 10, 2.3%), and others (*n* = 104, 24.1%). The remaining 26 children had no psychiatric disorder.

### Total score of the new questionnaire

The mean total score in the combined sample was 26.0 ± 4.2 (range from 19 to 40). For the clinical and community samples, the mean total score was 26.7 ± 4.2 (range from 19 to 40) and 24.2 ± 3.6 (range from 19 to 38), respectively. The clinical sample had a significantly higher score than the community sample (*p* < 0.001).

### Reliability

Cronbach’s alpha coefficients of the new questionnaire were 0.62 for the clinical sample, 0.65 for the community sample, and 0.65 for the combined sample. All of these values indicate acceptable (0.6 ≤ *α* < 0.7) internal consistency according to Cortina et al. [15].

The item-total correlation showed that the correlation of each item with the total scores calculated from the remaining items of the new questionnaire was significant except for “Child suddenly falls asleep in the middle of active behavior” (Table 1).

### Validity

Discriminant validity was investigated by comparing the total score of the new questionnaire between the children with sleep problems (CSHQ total score ≥ 41) and those without sleep problems (CSHQ total score < 41). Mean total score of the questionnaire was significantly higher in the children with sleep problems (27.2 ± 3.9 vs. 22.0 ± 2.1, *p* < 0.001).

Sensitivity and specificity were examined using ROC analysis (Fig. 1). Using the cutoff score of the original CSHQ as a threshold, the area under the curve was 0.89 (0.86–0.91) and the cutoff score was 24. Sensitivity was calculated to be 0.83 and specificity to be 0.78. The positive and negative predictive values were 92.3 and 59.1%, respectively. The proportion of children with a score above the cutoff with the new questionnaire was 68.4% in the combined sample and was significantly higher in the clinical sample than in the community sample (74.5 vs. 53.5%, *p* < 0.001).

Concurrent validity between the new questionnaire and the CSHQ for the total score in the combined sample was examined via correlation analysis using Pearson’s correlation coefficient. Both questionnaires showed a strong association for the total score (*r* = 0.81, *p* < 0.001) (Fig. 2). Total score of the new questionnaire was also significantly correlated with all CSHQ subscales: Bedtime Resistance, *r* = 0.39; Sleep Onset Delay, *r* = 0.29; Sleep Duration, *r* = 0.31; Sleep Anxiety, *r* = 0.46; Night Wakings, *r* = 0.32; Parasomnias, *r* = 0.65; Sleep Disordered Breathing, *r* = 0.37; and Daytime Sleepiness, *r* = 0.63 (all *p* < 0.001).

### Comparison with the model obtained by model selection

We also confirmed the reliability and validity of the model obtained from the CSHQ by model selection using stepAIC. The following 21 items were selected in the statistically reduced model: “Child falls asleep alone in own bed,” “Child falls asleep in parent’s or sibling’s bed,” “Child is ready to go to bed at bedtime,” “Child struggles at bedtime,” “Child is afraid of sleeping in the dark,” “Child is afraid of sleeping alone,” “Child sleeps the right amount,” “Child sleeps about the same amount each day,” “Child talks during sleep,” “Child is restless and moves a lot during sleep,” “Child moves to someone else’s bed during the night,” “Child snores loudly,” “Child awakens once during the night,” “Child wakes up by him/herself,” “Child wakes up in negative mood,” “Adults or siblings wake up child,” “Child has difficulty getting out of bed in the morning,” “Child has a good appetite in the morning,” “Child seems tired,” “Child has appeared very sleepy or fallen asleep while watching TV,” and “Child has appeared very sleepy or fallen asleep while riding in a car.” The Cronbach’s alpha coefficient of the statistically reduced model was 0.65. The sensitivity and specificity of the model as calculated by ROC analysis were slightly higher (0.85 and 0.92, respectively), and the correlation was stronger between the total scores of the model and the CSHQ (*r* = 0.92, *p* < 0.001) in comparison with the new questionnaire. By contrast, the correlation coefficients with each CSHQ subscale were within the same range as those of the new questionnaire but showed a different pattern, as follows: Bedtime Resistance, *r* = 0.63; Sleep Onset Delay, *r* = 0.35; Sleep Duration, *r* = 0.29; Sleep Anxiety, *r* = 0.55; Night Wakings, *r* = 0.53; Parasomnias, *r* = 0.57; Sleep Disordered Breathing, *r* = 0.25; and Daytime Sleepiness, *r* = 0.66 (all *p* < 0.001 by Pearson’s correlation coefficient).

## Discussion

We assessed the reliability and validity of a newly developed brief sleep questionnaire for children in a sample comprising both high-risk children and a community population. The items of the new questionnaire were selected from the CSHQ by clinical experts. The internal consistencies of the new questionnaire were acceptable for the community, clinical, and combined samples. Using the cutoff value of the CSHQ as a threshold, the new questionnaire was confirmed to have sufficient discriminatory power, and ROC analysis suggested similar sensitivity and specificity for the new questionnaire and the CSHQ. The new questionnaire also had strong correlations with the CSHQ and its subscales. In addition, we confirmed that the new questionnaire had similar reliability and screening ability compared with a statistically reduced model. These results show that the new questionnaire has utility similar to that of the CSHQ in screening for sleep problems in school-aged children.

A review of the available sleep questionnaires for children by Spruyt et al. [8] found that 57 instruments had been published as of 2011, with 22 of them suitable for school-aged children. The numbers of questionnaire items in these 22 instruments ranged from 6 to 67; 7 instruments had fewer than 20 items. Of these, 4 instruments focused on daytime sleepiness [19–22] and 1 instrument each focused on detection of snoring [23], morningness-eveningness chronotype [24], and aggression [25]. Including the instruments published after the review by Spruyt et al., no questionnaire with fewer than 20 items has focused on the screening of sleep problems in school-aged children.

Regarding reliability, the Cronbach’s alpha coefficient of our questionnaire was similar to that of the CSHQ reported in Owens et al. [9] for the community sample (0.65 vs. 0.68) but lower for the clinical sample (0.62 vs. 0.78). The clinical sample in Owens et al. was recruited from a pediatric sleep disorder clinic, whereas our study recruited participants from the patients of a pediatric psychiatric hospital. Because we aimed to analyze a clinical sample comprising children at high risk of but not diagnosed with sleep disorder, the alpha coefficient value of our clinical samples was pertinent. On the other hand, the sensitivity and specificity of the new questionnaire were similar to those of the CSHQ (sensitivity, 0.83 vs. 0.80; specificity, 0.78 vs. 0.70). Therefore, the screening ability of the new questionnaire appears to be similar to that of the CSHQ.

The reliability of the statistically reduced model obtained by model selection (stepAIC) was 0.65, and the sensitivity and specificity were 0.85 and 0.92, which were not clearly superior to those of the new questionnaire. Furthermore, the new questionnaire showed almost the same relationship with the subscales of the CSHQ. Our questionnaire comprises the items selected by clinical experts based on clinical importance. Therefore, the data suggest the satisfactory verification of the item selection of our questionnaire.

The 19 items of the new questionnaire were selected by multiple sleep medicine specialists and pediatric psychiatrists and were focused on the prevalence and importance of sleep disorders in a clinical setting. The new questionnaire contained 6 items excluded from the total score calculation of the CSHQ: “Child falls asleep with rocking or rhythmic movements,” “Child needs a special object in the room to fall asleep,” “Child resists going to bed at bedtime,” “Child wakes up very early in the morning,” “Child has a good appetite in the morning,” and “Child suddenly falls asleep in the middle of active behavior.” However, both questionnaires showed a strong correlation for the total score (*r* = 0.81, *p* < 0.001), and the total score of the new questionnaire also showed a significant positive correlation with all CSHQ subscales. Therefore, although our questionnaire does not include items used for calculation of the total and subscale scores of the original CSHQ, it should be able to detect children’s sleep problems covered by each subscale to some extent. The CSHQ also confirmed the interrelationships among subscales and showed that the eight subscales were not completely independent. Therefore, the new questionnaire may be able to screen for a similarly diverse range of sleep problems as the CSHQ.

The present study has several limitations. First, we did not use criteria for the diagnosis of sleep disorder because our clinical samples were not recruited from a pediatric sleep disorder clinic. Second, we used the CSHQ for children 6–12 years old and the CSHQ has not been validated in children ≥ 11 years old. However, there is no sleep questionnaire except the CSHQ suitable for use in children in this age range. Indeed, several studies have used the CSHQ for individuals > 10 years old [26, 27]. Therefore, we believe that it was significant that we could confirm the reliability and validity of the new questionnaire in a sample including 10–12-year-old participants. Third, the total mean score of the CSHQ in the elementary school children of this study (42.9) was higher than the cutoff score (41). Indeed, the mean score was comparable to that of studies with a relatively large number of community samples; the reported total mean scores of the CSHQ ranged from 38.7 to 47.0 in Western countries [28–31] and from 42.11 to 45.72 in Asian countries including Japan [27, 31, 32]. As shown above, the high prevalence of sleep problems using the CSHQ is universal among modern societies, not only in Japan. Another possible reason is that the cutoff used in this study (CSHQ total score ≥ 41) was established with American children aged 4–10 years [9] and may not suitable for children in countries with different common sleep habits such as co-sleeping [32, 33]. Further studies may be needed to revisit the cutoff score of the CSHQ in each country.

## Conclusions

We developed a brief sleep questionnaire consisting of 19 items selected based on clinical importance and confirmed its reliability and validity in children from both a high-risk population and a community population, as well as similar sensitivity and specificity compared with CSHQ. The new questionnaire is a simpler way to screen for sleep problems in school-aged children and detect sleep disorder at an earlier stage.

## Abbreviations

AIC: Akaike’s information criterion
CSHQ: Children’s Sleep Habits Questionnaire
ROC: Receiver operator characteristic
